# Integration and activity of hospital-based palliative care consultation teams: the INSIGHT multicentric cohort study

**DOI:** 10.1186/s12904-017-0209-9

**Published:** 2017-05-30

**Authors:** Pascale Vinant, Ingrid Joffin, Laure Serresse, Sophie Grabar, Hélène Jaulmes, Malika Daoud, Gabriel Abitbol, Pascale Fouassier, Isabelle Triol, Sylvie Rostaing, Marie-Dominique Brette, Isabelle Colombet, the INSIGHT investigators, Anne Abel, Gabriel Abitbol, Anne Bauby, Bénédicte de Corbière, Marie-Dominique Brette, Nathalie Chaillot, Malika Daoud, Barbara Edda-Messi, Adeline Ferry, Pascale Fouassier, Pascale Gauthier, Marie-Yvonne Guillard, Hélène Jeaulmes, Charles Jousselin, Michele Levi-Soussan, Didier Mercier, Vincent Montheil, Nathalie Moreau, Delphine Prenat-Molimard, Véronique Morize, Yolaine Raffray, Pascale Tocheport, Emilie Trabuc, Dominique Varin, Fabienne Weiler

**Affiliations:** 1 Hôpitaux Universitaires Paris Centre, Paris, France; 2 Hôpitaux Universitaires Paris Seine Saint-Denis, Paris, France; 3 Hôpitaux Universitaires La Pitié-Salpêtrière – Charles-Foix, Paris, France; 4 Public Health, Univ Paris Descartes, Paris, F-75006 France; 5 Hôpitaux Universitaires Paris Ouest, Paris, France; 6 Hôpitaux Emile Roux, Paris, France; 7 Hôpitaux Universitaires Paris Sud, Paris, France; 8 Hôpitaux Universitaires Est Parisien, Paris, France; 9 Hôpital Cochin, Unité Mobile de Soins Palliatifs (Bâtiment Copernic), 123 bvd de Port Royal, Cedex 14, 75679 Paris, France

**Keywords:** Palliative care, Health services evaluation, Multicenter study, Cohort study

## Abstract

**Background:**

Hospital-based Palliative Care Consultation Teams (PCCTs) have a consulting role to specialist services at their request. Referral of patients is often late. Early palliative care in oncology has shown its effectiveness in improving quality of life, thereby questioning the “on request” model of PCCTs. Whether this evidence changed practice is unknown. This multicentre prospective cohort study aims to describe the activity and integration of PCCTs at the patient level.

**Methods:**

For consecutive patients newly referred to participating PCCTs, the team collected the following data: circumstances of first referral, problems identified, number of interventions, patient’s survival after first evaluation and place of death.

**Results:**

Seventeen PCCTs based in university hospitals in Paris area, recruited 744 newly referred adult patients, aged 72 ± 15 years, 52% males, and 504(68%) with cancer as primary diagnosis. After 6 months, 548(74%) had died. At first evaluation, 12% patients were outpatients, 88% were inpatients. Symptoms represented the main reasons for referral and problems identified; 79% of patients had altered performance status; 24% encountered the PCCT only once. Median survival (1st-3rd quartile) after first evaluation by the PCCT was 22 (5–82) days for overall patients, and respectively 31 (8–107) days and 9 (3–34) days for cancer versus noncancer patients (p < 0.0001). Place of death was acute care hospital for 51.7% patients, and home or Palliative Care Unit for 35%. Patients referred earlier died more often in PCU.

**Conclusion:**

The study provides original data showing a still late referral to the PCCTs in France. Cancer patients represent their predominant activity. The integrated palliative care model seems to emerge besides the “on request” model which originally characterised their missions.

**Electronic supplementary material:**

The online version of this article (doi:10.1186/s12904-017-0209-9) contains supplementary material, which is available to authorized users.

## Background

In France, the first Palliative Care Consultation Team (PCCT) was created in 1989, shortly after the opening of the first inpatient Palliative Care Unit (PCU). Since then, the number of hospital-based teams has continuously increased. In 2013, the National End of Life Observatory counted 430 PCCT and 127 PCU all around the country. This high number of PCCTs is the result of the national policy for palliative care development whose main objective has been to disseminate palliative culture in hospital services concerned with end of life care. However, several studies have been published since 2008, showing the poor results of this policy. In a large population of patients who died in 200 acute care hospitals, only 20% of those whose death was deemed predictable received palliative care by a PCCT [1]. Death is still perceived by health professionals as an incongruity, a failure, and, as such, is largely hidden [2]. A national survey of doctors who signed death certificates also confirmed the delay in the appropriation of good decision-making practices at the end of life [3]: 30% of decisions taken for discontinuation or intensification of treatments before death were not discussed with patients themselves although they would have been capable for it. The more recent report, delivered to the French President for revision of the law on end of life [4] pointed the poor integration of palliative care in curative medicine with elusion of death by patients and professionals.

Yet, PCCTs are on first line to acculturate health professionals to palliative care. They have a consulting role, but usually advise specialist services only at their request. [5] They also have educational activities in order to raise awareness of palliative care needs of patients throughout the hospital. Systematic reviews, which suggested their efficacy to improve symptoms control, quality of life and family satisfaction with care, also underlined the heterogeneity original studies which limited their conclusions [6, 7]. One of the main difficulties encountered by PCCTs is late referral of patients: median reported times from first consultation to death vary between 6 and 11 days [8, 9]. While called lately, the PCCTs have a too short margin of action to prevent suffering and to anticipate the trajectory of care [10]. Some reasons described to explain this timing are the fear of harming the patient, the will of maintaining hope, the uncertainty of prognosis estimate, the insufficient communication skills [11, 12] and the lack of common referral criteria [13]

Temel et al. experimented an organization of systematic referral to palliative care, with first meeting of the patient with the PCCT shortly after the diagnosis of incurable lung cancer, and at least monthly meetings thereafter, integrated with standard oncologic care, until death [14]. This new model of early palliative care has been further evaluated by several randomised clinical trials in advanced cancer populations and has shown its effectiveness in improving quality of life [15–17]. The extent to which this evidence has impacted the practice of referral to specialist palliative care services is unknown.

Whereas some studies have described quantitative and qualitative activity of PCCT in various countries [9, 18, 19], published data describing this activity in the context of French healthcare system are sparse. In a previous study, we reported the six years’ experience and activity profile of a team which is based in a university hospital [20]. We still lack a more accurate insight into the activity and integration of PCCTs, from a broader sample of teams.

The objective of this multicentre prospective study was to describe the patients included by 17 palliative care consultation teams, to report the circumstances of their first referral, the problems identified by the team, its follow up activity, and the patients’ survival time after referral.

## Methods

### Population and collected data

This prospective cohort study has been conducted by the College of Palliative Care Physicians of the Assistance Publique – Hôpitaux de Paris, which run 33 acute care or long term care hospitals in Paris area, 25 of them having a PCCT. All physicians registered to be responsible for one of the 25 adult PCCTs have been invited for participation. Responding teams completed some details on the composition of its staff and the number of inpatients decedents per year in their hospital.

Participant teams included all patients consecutively newly referred and clinically evaluated between Oct 5 and Dec 6, 2014. At baseline, the team collected patient descriptive data (age, gender, main pathology, prognostic parameters), and described the circumstances of its first intervention and the problems identified during the intervention. The circumstances of intervention included its setting (short term or long term inpatient care, outpatient clinics or outpatient services), the reasons for referral and the service requesting consultation. Reasons for referral and problems identified by the team were collected using a common standard format, inspired from Sasahara et al. [19] and composed with: physical symptoms (pain, dyspnea, …), psychological symptoms, adaptation of symptomatic treatment, social issues, ethical questions, problems related to information of the patient or his/her family, decision for place of care, early encounter with PCCT (even without any appealing symptoms).

Six month after enrolment of the last patient, the PCCT collected data relative to its subsequent activity with each patient: number of interventions, interviews with family, referral to home palliative care services, follow up activity after the initial intervention(s), and discharge to a PCU. “Follow up activity” refers to either ongoing follow up by the team, or possible subsequent interventions upon repeated requests from services. Finally, vital status was recorded with, if applicable, the date and place of death.

The questionnaire used for data collection was developed by a working group composed of 8 doctors of the college, adapted from our previous study [20] and from the format used by Sasahara et al. [19] for the description of reasons for referral and identified problems. It has been tested for a week by all participating teams before the start of the study and subsequently reviewed for readability and face validity. A user guide was written with the help of a team, who had not participated in its design.

### Statistical analyses

Descriptive statistics were performed for all collected data, by frequencies and proportions for nominal variables, excluding missing data, and by mean (SD) or median (1st-3rd quartiles) for continuous variables, as appropriate to their distribution profile. Descriptions are provided according to the primary diagnosis dichotomised in cancer diagnosis, the most frequent, and other diagnosis

We described the activity of PCCTs, on the basis of the reasons for referral and the problems identified as completed for each patient by the PCCT right after its first evaluation. We determined the percentages of each item for all patients included in the study, by subgroup of cancer versus non cancer patients and by subgroup of patients evaluated by PCCT within their 7 last days of life versus before their 30 last days. For simplicity of presentation, we only report the 7 most frequent items. Detailed results are made available as supplement tables on the journal website.

We analysed the survival time after the first intervention of PCCT, with the Kaplan Meier method. Survival estimates between cancer patient and non-cancer patients were compared with the LogRank test.

In order to allow comparisons with other studies [20] and to produce an estimate that could be easily measurable by any PCCT, we also computed in the group of decedents the median time between the first referral to PCCT and death. The association of this indicator with the place of death was tested, using Chi^2^ test.

## Results

### PCCT characteristics and patients flow

Out of 25 PCCT of APHP, seventeen PCCTs agreed to participate in the study. All were based in university hospitals for adult patients. Four of these hospitals were specialized in geriatrics and provided long term inpatient care. The number of inpatient deaths recorded by each centre in 2014 ranged from 134 to 1384. The number of PCCT members ranged from 0.3 to 2.8 full-time equivalent physicians, and from 0.3 to 4 FTE nurses. The median number of new patients referrals to the PCCT in 2014 was 272 (range 95–452). The PCCTs included 744 patients and the median number (1^st^-3^rd^ quartiles) of patients included per PCCT was 46 (25–55).

### Patients’ characteristics and circumstances of the first intervention of PCCT

Table 1 summarizes the characteristics of all the 744 patients and the circumstances of the first intervention of the PCCT. These patients were 52% male and aged 72.4 years on average. Only 12% of patients were ambulatory at the time of the first referral to the PCCT (either evaluated in outpatient clinic or in medical consultation setting). Most patients were hospitalized (77% in acute care unit) and their first evaluation by PCCT which occurred at a median of 7 days after admission. Almost 70% of patients were referred by oncology, hematology and radiotherapy, hepato-gastroenterology, geriatrics or internal medicine staff.

**Table 1:** Patients’ characteristics and circumstances of the first evaluation by Palliative Care Consultation Team (*N* = 744)

	N	(%)
Men	385	(51.7)
Age, *mean (SD)*	*72.4*	*(15.1)*
Setting		
Short term inpatient unit	570	(77.0)
Time from admission (days), *median (Q1-Q3)*	*7*	*(3–16)*
Outpatient clinic	57	(7.7)
Medical consultation	33	(4.5)
Rehabilitation services	71	(9.6)
Long term inpatient unit	9	(1.2)
Primary diagnosis *(n = 739)* ^a^		
Cancer	504	(68.2)
Polypathology	70	(9.5)
Cardiovascular	39	(5.3)
Neurovascular	30	(4.1)
Other neurologic	27	(3.7)
Respiratory	20	(2.7)
Renal	12	(1.6)
Other	37	(5.0)
Service requesting consultation *(n = 723)* ^a^		
Oncology/Haematology/Radiotherapy	167	(23.1)
Gastroenterology and hepatology	130	(18.0)
Geriatrics	129	(17.8)
Internal Medecine	77	(10.7)
Pneumology	68	(9.4)
Neurology	29	(4.0)
Cardiology	29	(4.0)
Other	94	(13.0)
Prognostic factors		
PS^b^ ≥ 3 and/or Karnofsky ≤ 50 *(n = 681)* ^a^	538	(79.0)
Albumin, g/L, *median (Q1-Q3) (n = 367)* ^a^	*27*	*(23–33)*
Bed sores	95	(12.8)
Swallowing disorders	201	(27.0)

^a^after excluding missing data

^b^Performance Status, as measured by the Eastern Cooperative Oncology Group (ECOG) scale (http://www.ecog.org/general/perf_stat.html)

Most patients had an impaired general condition, as reflected by prognostic parameters: 79% had a an ECOG performance status ≥3 or a Karnofski < 50%, 27% had some swallowing disorders, 13% had bed sores.

A majority of patients (68%) had cancer as primary diagnosis. Table 2 summarizes their characteristics and cancer history at the time of first PCCT intervention: 62% of them had 2 or more metastatic sites and 37% had no anticancer treatment (discontinued or never started).

**Table 2:** CANCER patients’ characteristics and circumstances of the first evaluation by Palliative Care Consultation Team (*N* = 504)

	Number	Percent
Men	277	(55.6)
Age, *mean (SD)*	*68.6*	*(14.2)*
Setting		
Short term inpatient unit	386	(76.6)
Rehabilitation services or long term unit	31	(6.2)
Outpatient clinic	55	(10.9)
Medical consultation	32	(6.3)
Service requesting consultation	*(N = 493)* ^a^	
Oncology	141	(28.6)
Gastroenterology and hepatology	119	(24.1)
Pneumology	56	(11.4)
Geriatrics	50	(10.1)
Internal Medecine	36	(7.3)
Neurology	17	(3.4)
Haematology	15	(3.0)
Dermatology	14	(2.8)
Radiotherapy	7	(1.4)
Others	17	(14.0)
Cancer history		
Incurable at the initial diagnosis *(N = 371)* ^a^	217	(58.5)
Metastatic sites	*(N = 393)* ^a^	
0	45	(11.5)
1	104	(26.5)
2	115	(29.3)
3 or more	129	(32.8)
Current evolution of disease	*(N = 456)* ^a^	
At diagnosis (treatment not started)	47	(10.3)
Stability or answer to treatment	35	(7.6)
Progression (local or metastatic)	374	(82.0)
Antitumoral treatment was :	*(N = 433)* ^a^	
Not yet or never started	108	(24.9)
Continuing	166	(38.3
Discontinued	159	(36.7)
Prognostic factors		
PS^b^ ≥ 3 and/or Karnofski ≤ 50 *(n = 474)*	336	(70.9)
Albumin, g/L, *median (Q1-Q3) (n = 246)*	*29*	*(23–33)*

^a^after excluding missing data

^b^Performance Status, as measured by the Eastern Cooperative Oncology Group (ECOG) scale (http://www.ecog.org/general/perf_stat.html)

### Reasons for referral and problems identified by PCCT

The major reasons for referral were: pain, 375 (50.4%); early encounter with PCCT, 171 (23%); the decision for place of care, 147 (19,8%); a decision to withhold or withdraw treatments, 128 (17.2%). The major problems identified by PCCT were pain 420 (56.5%), fatigue 246 (33.1%), anxiety/depression, 223 (30%), choice of drug/change in the drug dosage or the route of administration, 191 (25.7%), dyspnea/cough/sputum, 191(25.7%). (see Additional file 1)

The main problems identified by the PCCT and not mentioned as a reason for referral were among physical and psychiatric/emotional issues: constipation, appetite loss/difficulty of oral intake, fatigue, insomnia (see Additional file 1)

Table 3 summarizes the most frequent reasons for referral and problem identified by subgroups of 504 patients with cancer and 240 patients with other primary diagnosis. In cancer patients, the first two reasons for referral were pain (57.3%) and early encounter with PCCT (24.4%), whereas, in the other subgroup, pain remained the main reason for referral (36%), but the second one was decision to withhold or withdraw treatments (34%).

**Table 3:** Seven most frequently reported reasons for referral and problems identified by PCCT, by subgroup of patients with CANCER or OTHER primary diagnosis

Cancer Patients (*n* = 504)			Patients with other primary diagnosis (*n* = 240)		
Reasons for referral					
	n	(%)		n	(%)
Pain	289	(57,3)	Pain	84	(35,7)
Early encounter	123	(24,4)	Decision to withhold or withdraw treatments	79	(33,6)
Decision for place of care	93	(18,5)	Decision for place of care	54	(23,0)
Anxiety/depression/emotional distress	67	(13,3)	Dyspnea/cough/sputum	49	(20,9)
Dyspnea/cough/sputum	57	(11,3)	Early encounter	46	(19,6)
Decision to withhold or withdraw treatments	47	(9,3)	Anxiety/depression/emotional burden/grief, as a family issue	33	(14,0)
Anxiety/depression/emotional burden/grief, as a family issue	35	(6,9)	Swallowing disorders	25	(10,6)
Problems identified by PCCT					
Pain	311	(61,7)	Pain	106	(45,1)
Fatigue	185	(36,7)	Decision to withhold or withdraw treatments	101	(43,0)
Anxiety/depression/emotional distress	170	(33,7)	Dyspnea/cough/sputum	80	(34,0)
Early encounter	130	(25,8)	Decision for place of care	69	(29,4)
Appetite loss / difficulty of oral intake	122	(24,2)	Choice of drug/change in the drug dosage or the route of administration	68	(28,9)
Choice of drug/change in the drug dosage or the route of administration	120	(23,8)	Anxiety/depression/emotional burden/grief, as a family issue	61	(26,0)
Decision for place of care	117	(23,2)	Fatigue	59	(25,1)

The same data are described for decedent patients referred more than 30 days (171 patients) and for those referred less than 7 days before death (198 patients). Pain is always the major reason for referral. For the later, the most frequent reasons roughly match the problems identified by PCCT, which does not seem to be the case for patients referred earlier, except for pain and early encounter of PCCT (see Table 4).

**Table 4:** Seven most frequently reported reasons for referral and problems identified by PCCT, by subgroup of decedents patients referred more than 30 days or less than 7 days before death

Patients referred < 7 days before death (*n* = 198)			Patients vu > 30 days before death (n = 171)		
Reasons for referral					
	n	(%)		n	(%)
Pain	79	(39,9)	Pain	97	(56,7)
Dyspnea/cough/sputum	53	(26,8)	Early encounter	52	(30,4)
Decision to withhold or withdraw treatments	45	(22,7)	Choice of drug/change in the drug dosage or the route of administration	25	(14,6)
Decision for place of care	44	(22,2)	Dyspnea/cough/sputum	19	(11,1)
Choice of drug/change in the drug dosage or the route of administration	33	(16,7)	Decision for place of care	18	(10,5)
Early encounter	31	(15,7)	Decision to withhold or withdraw treatments	17	(9,9)
Anxiety/depression/emotional burden/grief, as a family issue	24	(12,1)	Anxiety/depression/emotional distress	15	(8,8)
Problems identified by PCCT					
Pain	104	(52,5)	Pain	94	(55,0)
Dyspnea/cough/sputum	87	(43,9)	Fatigue	68	(39,8)
Decision to withhold or withdraw treatments	59	(29,8)	Early encounter	59	(34,5)
Choice of drug/change in the drug dosage or the route of administration	59	(29,8)	Anxiety/depression/emotional distress	51	(29,8)
Anxiety/depression/emotional burden/grief, as a family issue	56	(28,3)	Appetite loss / difficulty of oral intake	49	(28,7)
Fatigue	54	(27,3)	Choice of drug/change in the drug dosage or the route of administration	43	(25,1)
Anxiety/depression/emotional distress	50	(25,3)	Anxiety/depression/emotional burden/grief, as a family issue	38	(22,2

### Follow up activity and survival after 1st intervention

Table 5 presents the follow up data for the whole study population and for subgroups of cancer and non-cancer patients. Among all patients included, 177 (24.1%) had only one interview with the PCCT and 13% had more than 10. However, 155 (20.8%) patients had no further follow up activity after initial intervention(s) of the PCCT. Only 10% were referred to home palliative care services.

**Table 5:** Subsequent activity and follow up data, according to primary diagnosis

Patients interviews with PCCT	Total (*N* = 744)		Cancer (*N* = 504)		Other (*N* = 235)	
	N	(%)^a^	N	(%)^a^	N	(%)^a^
	(*n* = 734)		(*n* = 498)		(*n* = 233)	
Once	177	(24.1)	110	(22.1)	66	(28.3)
2 to 5	340	(46.3)	220	(44.2)	119	(51.1)
6 to 10	121	(16.5)	90	(18.1)	30	(12.9)
11 or more	96	(13.1)	78	(15.7)	18	(7.7)
Interviews with relatives	398	(54.7)	280	(56.6)	118	(51.5)
Referral to home care services	64	(9.0)	54	(9.9)	10	(4.4)
Discharge to PCU	184	(32.5)	136	(36.6)	46	(24.2)
No follow up activity after initial interventions^c^	155	(20.8)	112	(22.5)	42	(17.9)
Survival after 1st intervention of PCCT (days)^b^						
median (Q1-Q3)	22	(5–82)	31	(8–107)	9	(3–34)
Rate of death,% (± SD) at 3 days	18.0	(1.5)	14.5	(1.6)	25.9	(2.3)
at 7 days	30.3	(1.8)	23.4	(2.0)	45.1	(3.4)
at 30 days	57.1	(2.0)	49.9	(2.4)	72.7	(3.2)
Number of decedents at the end of follow up	548	(73.7)	358	(71.0)	186	(79.1)
Place of death	(*n* = 543)		(*n* = 354)		(*n* = 185)	
Acute care hospital	259	(47.9)	169	(47.7)	90	(48.6)
Emergency room	9	(1. 7)	4	(1.1)	5	(2.7)
Intensive care unit	10	(1.9)	3	(0.8)	7	(3.8)
Private hospital	3	(0.6)	3	(0.8)	0	-
Total acute care hospital	281	(51.7)	179	(50.6)	102	(55.1)
Rehabilitation center	60	(11.1)	32	(9.0)	28	(15.1)
Long term care hospital	5	(0.9)	1	(0.3)	3	(1.6)
PCU	163	(30.1)	120	(33.9)	41	(22.2)
Home or retirement home	25	(4.8)	20	(5.7)	5	(2.7)
Other or unknown	8	(1.5)	2	(0.6)	6	(3.2)
When deceased at hospital^d^,						
Patient already knew the staff	172	(57.5)	111	(63.4)	60	(49.2)
Patient was referred to PCCT during his/her last stay	266	(87.5)	145	(80.5)	119	(97.5)
When deceased in PCU	(*n* =162)		(*n* = 121)		(*n* = 39)	
Time from admission to death (days)*, median (Q1-Q3)*	*9*	*(5–18)*	*11*	*(5–21)*	*7*	*(4–11)*
Death within 3 days after admission	29	(17.8)	23	(19.0)	6	(15.4)

*PCU* palliative care unit, *PCCT* palliative care consultation team

^a^unless otherwise mentioned, percentages are computed after excluding missing data

^b^median of survival and death rates are estimated by Kaplan Meier method

^c^“Follow up activity after initial intervention(s)” refers to either ongoing follow up by the team, or possible subsequent interventions upon repeated requests from services

^d^“hospital” as a place of death include acute care hospital, rehabilitation center and long term care hospital

Kaplan-Meier survival curves after the first referral to PCCT are shown in Figure. Overall, patients died in median 22 days (IQR: 5–82) after referral to PCCT. Median survival was 31 days (IQR: 8–107) for patients diagnosed with cancer and 9 days (IQR 3–34) for patients with other primary diagnosis (log-rank p < 0.0001). Among all included patients, 18% first met the PCCT within 3 days before death, and 57.1% within 30 days before death. The place of death was acute care hospital for 281 (51.7%) patients, home or retirement home for 25 (4.6%), and palliative care unit for 163 (30%).

For 88% of patients who deceased at hospital, the first referral to PCCT occurred during the last stay and for 18% of patients who deceased in a Palliative Care Unit (PCU), death occurred within 3 days after their admission.

In the subgroup of 541 decedent patients, the median (1^st^-3^rd^ quartile) number of days from first referral to death, was 15 (5–40). It was respectively 21.5 (6–51) and 7 (3–20.3) for the 352 decedents of the cancer group and the 185 decedents of the non cancer group,

Patients referred earlier died significantly more often at home or in PCU as compared with patients referred later: 90 (53%), 81 (48%) and 12 (6%) (p < 0.0001) respectively for patients referred before 30 days, between 30 and 7 days, and less than 7 days before death.

## Discussion

Our study provides for the first time some original multicentre data describing detailed activity of PCCTs based in French university hospitals. This activity is predominantly oriented towards cancer patients (68% of study population). The most frequent reasons for referral are pain, early encounter with PCCT and decision of the place of care, whereas the main problems identified by the PCCTs are symptoms (pain, fatigue, anxiety and depression, respiratory symptoms). A large majority of patients are referred to palliative care when their performance status is already altered and late in their trajectory of care, with an overall 22 days median survival after the first evaluation by the PCCT and mainly during their last hospital stay. Estimated only in decedent patients identified by PCCT, the median time from first referral to death was 15 days. However, 38% of cancer patients are often referred while still receiving anticancer treatment, and significantly earlier than patients with other primary diagnosis, suggesting an emerging integration of the recent concept of “early palliative” care recommended in oncology [21, 22]. Our results also suggest that the early referral to PCCT has some impact on the trajectory of care, allowing that more patients die at home or in PCU.

Although 17 different teams participated to our study, the recruited population may not be representative of all French PCCTs activity. The participant teams are all based in university hospitals lead by the main health organisation of Paris area (*Assistance Publique-Hôpitaux de Paris*).

The distribution of location of death in our study population is also interesting to compare to national statistics obtained from death certificates. Indeed, we found in our study that only 3% of decedents died at home, whereas this proportion is 26% in national data [23]. This national statistics is influenced by age, cause of death and density of health care services: the proportion of subjects dying at home is higher in elderly people, dying from cardiovascular disease or violent causes (>30%) whereas it is lower in people dying from cancer (19%), and in urban areas with high density of hospitals supply. Our results are obtained from an inpatients cohort and confirm that patients who access palliative care through hospital based PCCTs are younger on average and have predominantly cancer disease.

In an analysis of national hospital activity data, the place of death of cancer inpatients is PCU for 10%, intensive care unit for 10%, in acute care units for 64% [24]. The three-fold proportion of death that we found in our population (34% of cancer patients deceased in PCU) can be partly explained by the high number of PCUs concentrated in Paris area (375 beds in the 1175 at the national level in 2010) [25].

The timing of referral to PCCT observed in the whole study population is late, as reported in other studies, whether retrospectively described from decedents series [8, 20], or prospectively estimated by survival analysis [9]. Even though our method for data collection allowed us to perform survival analyses, we also provide an estimate based on the decedent follow back method, which may be more feasible for teams to reproduce in their practice. In the subgroup of patients referred within their 7 last days of life, the problems identified by PCCTs seem to be in agreement with the reasons for referral, suggesting that referring staff have integrated what specialist palliative care can bring to help patient’s end-of life care. Conversely, the reasons why requesting advice earlier in the trajectory of care seem to be less clear, as reflected by a lower agreement between reasons for referral and problems identified by the PCCT. Moreover the reason for referral invoked by clinicians is “early encounter with PCCT” for 16% of patient referred less than 7 days before death. This reflects the lack of prognostic skills, although lots of factors and scores have been validated [26–29]. In the large subgroup of cancer patients (68%), the PCCT is introduced earlier in the trajectory or care. However, with a median survival time of 31 days after first intervention of PCCT, we are far from the model of early palliative care described and evaluated in clinical trials by Temel et al. [15] and Zimmermann et al. [16]. In this model, a first consultation and follow up of specialist palliative care is offered shortly after the diagnosis of advanced cancer, in parallel with standard oncologic care. Both trials have been performed in specialist cancer centres. In our setting, only 29% of cancer patients are referred by oncology wards, others being referred by other medical specialties where cancer patients represent a fraction of recruitment. An effective integrated onco-palliative care model may be more difficult to implement in such setting, with multiple care teams. Such integrated palliative care model actually marks a significant change of paradigm from the original missions of PCCTs, defined as counselling.

Our results show that they developed two different patterns of activity: a consulting activity, giving advice to health professionals without necessity to follow up the patient, and a more structured activity based on stronger collaboration with referring staff and regular follow up of patients until death. In this last type of activity, the request is still late, whereas there is strong evidence that patients can benefit from earlier introduction of palliative care [15, 16].

## Conclusions

The study provides original data allowing appreciation of the reality of PCCTs’ activity in France. Cancer patients represent their predominant activity. There is a need to extend palliative care to other chronic diseases. The timing of referral to the PCCTs is late. However, referring staff correctly identify the needs of patients in their last 7 days of life, so that the referral to PCCT is appropriate. The most frequent needs are the relief of pain or other symptoms and decisions on withdrawing or withholding treatments. For cancer patients as compared to patients with patients with other chronic diseases, the timing of referral is earlier. However, considering the evidence and strong recommendation of the early palliative care model in oncology, this timing remains late. Our results only suggest that the integrated palliative care model is emerging besides the “on request” model of referral to PCCTs which originally characterised their missions. This evolution appeals for a formal revision of these missions beyond the acculturation of health professionals to palliative care. The resources allocated to palliative care services should be adjusted consequently. In oncology, efforts should concentrate on educating patients and oncologists to promote the integrated onco-palliative care model. The evidence base for this model has been established in the setting of North American Healthcare system, first showing its feasibility and then, testing its effectiveness. Therefore, its implementation in the context of other countries must be supported by a research focused first on the evaluation of its feasibility, as a required condition for an actual change of practice.

## Additional file

Additional file 1: Table S1. Most frequently reported reasons for referral and problems identified by the PCCT in the whole population (744 patients). **Table S2.** Number of patients with item selected as reason for referrals (R), identified problem (P), both (B) and the proportion of problem identified without having been mentioned as a reason for referral ((P-B) / P). Total number of patients is *n* = 744. (DOCX 36 kb)

## Abbreviations

IQR: Interquartile Range
PCCT: Palliative Care Consultation Team
PCU: Palliative Care Unit
SD: Standard Deviation
