# Supplementary material for: Integration and activity of hospital-based palliative care consultation teams: the INSIGHT multicentric cohort study
Source: BMC Palliat Care. 2017 May 30;16:36. doi: 10.1186/s12904-017-0209-9 (PMC5450075; doi:10.1186/s12904-017-0209-9)
Supplement: Additional file 1: Table S1. — Most frequently reported reasons for referral and problems identified by the PCCT in the whole population (744 patients). Table S2. Number of patients with item selected as reason for referrals (R), identified problem (P), both (B) and the proportion of problem identified without having been mentioned as a reason for referral ((P-B) / P). Total number of patients is n = 744. (DOCX 36 kb) [file 12904_2017_209_MOESM1_ESM.docx]

**Table Supplement 1: Most frequently reported reasons for referral and problems identified by the PCCT in the whole population (744 patients)**

| **Reasons for referral** | N | % |  | **Problems identified** | N | % |
| --- | --- | --- | --- | --- | --- | --- |
| Pain | 375 | 50,4 |  | Pain | 420 | 56,5 |
| Early encounter | 171 | 23,0 |  | Fatigue | 246 | 33,1 |
| Decision for place of care | 147 | 19,8 |  | Anxiety/depression/emotional distress | 223 | 30,0 |
| Decision to withhold or withdraw treatments | 128 | 17,2 |  | Choice of drug/change in the drug dosage or the route of administration | 191 | 25,7 |
| Dyspnea/cough/sputum | 109 | 14,7 |  | Dyspnea/cough/sputum | 191 | 25,7 |
| Choice of drug/change in the drug dosage or the route of administration | 107 | 14,4 |  | Decision for place of care | 186 | 25,0 |
| Anxiety/depression/emotional distress | 88 | 11,8 |  | Early encounter | 180 | 24,2 |
| Anxiety/depression/emotional burden/grief, as a family issue | 68 | 9,1 |  | Decision to withhold or withdraw treatments | 163 | 21,9 |
| Fatigue | 60 | 8,1 |  | Appetite loss / difficulty of oral intake | 161 | 21,6 |
| Other physical symptoms | 45 | 6,0 |  | Anxiety/depression/emotional burden/grief, as a family issue | 160 | 21,5 |

**Table Supplement 2: Number of patients with item selected as reason for referrals (R), identified problem (P), both (B) and the proportion of problem identified without having been mentioned as a reason for referral ((P-B) / P). Total number of patients is n = 744.**

|  | **R** | **P** | **B** | **(P-B)/P (%)** |  |  | **R** | **P** | **B** | **(P-B)/P (%)** |
| --- | --- | --- | --- | --- | --- | --- | --- | --- | --- | --- |
| **Physical/pharmacological issues** |  |  |  |  |  | **Social Issues** |  |  |  |  |
| Pain | 375 | 420 | 306 | 27,1 |  | Absence of caregivers | 13 | 50 | 8 | 84,0 |
| Dyspnea/cough/sputum | 109 | 191 | 90 | 52,9 |  | Economic/work problem | 6 | 18 | 6 | 66,7 |
| Appetite loss / difficulty of oral intake | 39 | 161 | 30 | 81,4 |  | Relative in charge | 4 | 20 | 3 | 85,0 |
| Swallowing disorders | 41 | 93 | 32 | 65,6 |  | Native of foreign country | 4 | 16 | 4 | 75,0 |
| Fatigue | 60 | 246 | 48 | 80,5 |  | **Ethical issues** |  |  |  |  |
| Nausea/vomiting | 21 | 65 | 15 | 76,9 |  | Discontinuation of anticancer treatments | 30 | 40 | 19 | 52,5 |
| Abdominal swelling/ascites | 12 | 50 | 11 | 78,0 |  | Decision to withhold or withdraw treatments | 128 | 163 | 107 | 34,4 |
| Intestinnal obstructive symptoms | 11 | 30 | 9 | 70,0 |  | Involvement of double effect | 13 | 36 | 12 | 66,7 |
| Constipation | 9 | 84 | 6 | 92,9 |  | Performing a sedation | 6 | 15 | 4 | 73,3 |
| Edema/lymphedema | 10 | 38 | 7 | 81,6 |  | Request for euthanasia | 3 | **7** | 1 | 85,7 |
| Insomnia | 24 | 66 | 19 | 71,2 |  | Hasten death | 4 | 5 | 1 | 80,0 |
| Bed sores/open wound | 22 | 34 | 18 | 47,1 |  | Patient’s Information/  living wills | 0 | 53 | 0 | 0,0 |
| Other | 45 | 61 | 28 | 54,1 |  | Shared decision making process | 34 | 58 | 29 | 50,0 |
| Choice of drug/change in the drug dosage or the route of administration | 107 | 191 | 91 | 52,4 |  | **Decision for place of care** | 147 | 186 | 119 | 36,0 |
| **Psychiatric/emotional/spiritual issues** |  |  |  |  |  | **Early encounter** | **171** | **180** | 127 | 29,4 |
| Anxiety/depression/emotional distress. | 88 | 223 | 73 | 67,3 |  |  |  |  |  |  |
| Insomnia | 3 | 71 | 2 | 97,2 |  |  |  |  |  |  |
| Delirium | 35 | 85 | 31 | 63,5 |  | **Family issues** |  |  |  |  |
| Spirituel issues | 2 | 10 | 2 | 80,0 |  | Anxiety/depression/emotional distress | 68 | 160 | 60 | 62,5 |
| Other | 12 | 34 | 6 | 82,4 |  | Illness understanding/choice of treatment | 34 | **79** | 25 | 68,4 |
| **Diagnosis/treatment information issues** |  |  |  |  |  | Shortage of practical knowledge/skills | **7** | **24** | 4 | 83,3 |
| Illness understanding/choice of treatment | 35 | 110 | 30 | 72,7 |  | Integration of prognosis information | 33 | 93 | 24 | 74,2 |
| Anxiety about side effects anf anticancer treatment | 2 | 13 | 1 | 92,3 |  |  |  |  |  |  |
